# Optical coherence tomography analysis at initial diagnosis in patients with retinitis pigmentosa and associated cystoid macular edema

**DOI:** 10.1371/journal.pone.0325654

**Published:** 2025-06-25

**Authors:** Suk Hoon Jung, Sang Un Yi, Bo-Een Hwang, Young Gun Park, Young-Hoon Park

**Affiliations:** 1 Department of Visual Science and Ophthalmology, Seoul St. Mary’s Hospital, College of Medicine, The Catholic University of Korea, Seoul, Republic of Korea; 2 Catholic Institute for Visual Science, College of Medicine, The Catholic University of Korea, Seoul, Republic of Korea; Akita University: Akita Daigaku, JAPAN

## Abstract

This retrospective study analyzed optical coherence tomography (OCT) findings in 130 eyes of 130 patients with retinitis pigmentosa (RP) at initial diagnosis, including 42 with cystoid macular edema (CME) and 88 without, between September 2016 and March 2024. The CME group exhibited increased central macular thickness (CMT) (257.50 ± 104.98 µm vs. 171.40 ± 73.15 µm, p = 0.000), whereas the non-CME group had greater subfoveal choroidal thickness (SCT) (294.52 ± 122.85 µm vs. 246.98 ± 87.31 µm, p = 0.043), total choroidal area (TCA) (4.64 ± 1.98 mm² vs. 3.82 ± 1.34 mm², p = 0.031), stromal area (SA) (1.85 ± 0.76 mm² vs. 1.53 ± 0.54 mm², p = 0.025), luminal area (LA) (2.79 ± 1.22 mm² vs. 2.30 ± 0.81 mm², p = 0.033), and foveal avascular zone in the superficial capillary plexus (FAZ_SCP) (0.42 ± 0.31 mm² vs. 0.27 ± 0.12 mm², p = 0.022). The CME group had more moderate stage cases (47.62% vs. 26.14%, p = 0.015), while the non-CME group had more advanced cases (39.77% vs. 9.52%, p = 0.000). Visual acuity (logMAR) worsened in advanced stages for both groups (CME: 1.62 ± 0.79, p = 0.003; Non-CME: 1.12 ± 0.80, p = 0.000). In the CME group, FAZ in the deep capillary plexus (FAZ_DCP) enlarged from moderate to advanced stages (0.28 ± 0.12 mm² to 0.64 ± 0.09 mm², p = 0.025), and vessel density in the deep capillary plexus (VD_DCP) decreased from early to moderate stages (31.83 ± 3.94% to 28.75 ± 2.71%, p = 0.036), whereas superficial capillary plexus vessel density (VD_SCP) remained stable across stages (early: 32.82 ± 2.59%, moderate: 31.04 ± 2.37%, advanced: 31.52 ± 1.26%, all p > 0.1). The non-CME group exhibited progressive declines in CMT (early: 226.27 ± 38.60 µm, moderate: 195.04 ± 52.56 µm, advanced: 108.83 ± 59.72 µm, all p < 0.01) and choroidal vascularity index (CVI) (early: 0.61 ± 0.02, moderate: 0.60 ± 0.02, advanced: 0.58 ± 0.04, all p < 0.05). In the CME group, visual acuity (logMAR) was positively correlated with cyst area (p = 0.019, rho = 0.361) and FAZ_DCP (p = 0.002, rho = 0.564). These findings suggest that RP-CME may be associated with choroidal atrophy regardless of disease stage and could have a compensatory mechanism to SCP. Cyst area and FAZ_DCP may serve as indicators of visual acuity in RP-CME.

## Introduction

Retinitis pigmentosa (RP) is an inherited retinal dystrophy characterized by progressive degeneration of photoreceptors, primarily affecting rod cells [1,2]. This leads to symptoms such as night blindness, visual field defects, and varying degrees of vision loss, primarily affecting night and mid-peripheral vision, with gradual deterioration of central visual acuity that can progress to low vision or blindness [3]. Cystoid macular edema (CME) is a relatively common finding in patients with RP. The incidence of RP is about 1 in 4,000 people [4], and various studies report the occurrence of RP-CME to be around 10–50% [5,6]. Previous studies have proposed several hypotheses for the pathogenesis of CME in RP, including blood-retina barrier (BRB) breakdown, retinal pigment epithelium (RPE) pumping dysfunction, inflammatory responses, and vitreous traction [6,7]. CME can exacerbate central vision deterioration and profoundly impact patients’ quality of life, especially considering that most individuals with RP already have peripheral visual field loss [8]. Therefore, early detection and management of CME in RP patients may be a crucial issue. Thus, this study aimed to investigate the differences between the CME and non-CME groups by utilizing OCT (optical coherence tomography) and OCTA (optical coherence tomography angiography) images at the time of initial RP diagnosis, and to explore potential mechanisms underlying the development of CME. Furthermore, since no studies have included the CME group in stage-by-stage analyses of RP progression, this study aimed to perform such an analysis, incorporating the CME group.

## Materials and methods

### Study population, inclusion and exclusion criteria

This study was reviewed and approved by the Institutional Review Boards of the Seoul St. Mary’s Hospital, College of Medicine, The Catholic University of Korea (approval number: KC24RASI0401), and adhered to the tenets of the Declaration of Helsinki. The requirement for written informed consent was waived because of the retrospective nature of this study. Data were accessed for this study from June 23rd, 2024 to October 31st, 2024 in accordance with IRB approval. The data were anonymized by a designated data anonymization researcher after data collection, ensuring that individual patients could not be identified. The study involved 130 eyes from 130 patients of the Korean population who were initially diagnosed with RP at Seoul St. Mary’s Hospital between September 2016 and March 2024. RP was diagnosed based on the presence of peripheral bone spicule pigment migration into the retina, as identified by fundus examination, with a chart review of the patient’s clinical history, characteristic fundus appearance, visual field defect, electroretinography findings and genetic testing [9]. The stages of RP were classified according to the width of the inner segment ellipsoid zone on OCT images, as described by Oh R et al. [10]. Specifically, a width of ≥ 2500 μm was classified as early stage, < 2500 μm as moderate stage, and the absence of the ellipsoid zone as advanced stage. CME was defined as the presence of one or more fluid-filled, intraretinal cystoid spaces in the macula on OCT images [7]. If CME was present in both eyes, the eye with the greater central macular thickness (CMT) was selected to enable a clearer assessment of the relationship between CME severity and other clinical or OCT parameters. For unilateral CME, the affected eye was analyzed, and for patients without CME, the right eye was included in the analysis.

Exclusion criteria included the presence of other vitreoretinal disorders (such as epiretinal membrane, vitreomacular traction, diabetic retinopathy, retinoschisis, uveitis, age-related macular degeneration, retinal vascular diseases, etc.) and optic nerve disorders like glaucoma. Additionally, patients with high media opacity, high myopia (>6 D), recent ocular surgery within the past 6 months, or other corneal diseases were excluded. Those with a documented history of prior CME treatment at other hospitals were also excluded. Furthermore, cases with poor fixation or insufficient OCT image quality that impeded adequate visualization were excluded.

### Patient data collection

Detailed data collected included age, sex, lens status, presence of diabetes and hypertension, next generation sequencing (NGS)-based genetic test results, and best-corrected visual acuity (BCVA). OCT and OCTA images at the initial visit were analyzed using the Topcon DRI OCT Triton^®^. NGS testing was outsourced to the department of laboratory medicine, where DNA was extracted from peripheral blood and analyzed to identify genes with pathogenic or likely pathogenic variants, along with their inheritance patterns. These genetic test results were collected to investigate the potential association between specific genetic variants and RP progression or CME occurrence. Visual acuity was converted from Snellen to Logarithm of the Minimum Angle of Resolution (logMAR) for analysis [11]. In cases of low vision, based on the study by Baltinas J et al. [12], count fingers, hand movements, light perception, and no light perception were assigned logMAR values of 2.0, 2.3, 2.6, and 2.9, respectively, for evaluation and analysis.

### OCT and OCTA analyses of the retina and choroid

OCT examinations were performed between 08:30 and 17:30, and the OCT images analyzed in this study were those obtained at the time of initial RP diagnosis. A horizontal B-scan image centered on the fovea was selected using the Topcon DRI OCT Triton^®^, and central macular thickness (CMT), subfoveal choroidal thickness (SCT), and the width of the inner segment ellipsoid zone were manually measured using the Topcon IMAGEnet 6.0 software. CMT was defined as the distance from the internal limiting membrane (ILM) to the retinal pigment epithelium (RPE) [13], while SCT was defined as the distance from Bruch’s membrane to the sclerochoroidal junction [7,14].

Cyst area was defined as the total area of all intraretinal cysts within the macular region on OCT B-scan image. To calculate the cyst area in the macula from the OCT B-scan image, the boundaries of all cystoid spaces were traced using the freehand selection tool in ImageJ 1.53e software (National Institutes of Health, USA) and individually added to the Region of Interest (ROI) manager. Afterward, all cystoid spaces were combined using the ‘OR (combine)’ function, and the total cystoid space was measured.

**Fig 1:**
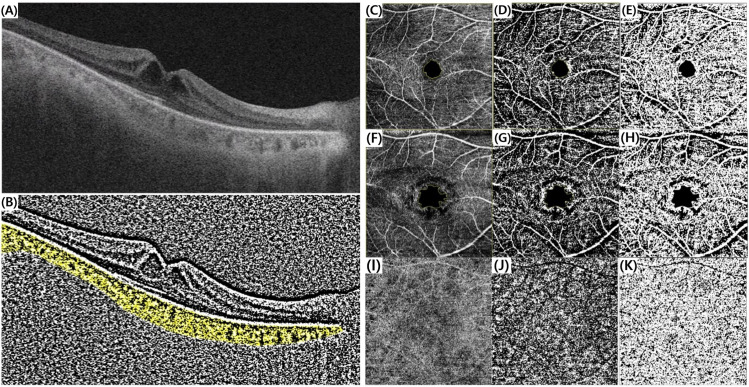
Parameter measurements using optical coherence tomography (OCT) images. (A) Swept-source optical coherence tomography (SS-OCT) image of the RP-CME patient. (B) To determine the total choroidal area, the binary version of SS-OCT images was generated using the Niblack auto-local threshold method, followed by applying the ‘Color Threshold’ tool to select pixels representing the luminal area. (C-E) Calculation of superficial capillary plexus (SCP) vascular density (VD) excluding the foveal avascular zone (FAZ) using the Otsu auto-local threshold. (C) Original image excluding the FAZ. (D) Binarized image of SCP excluding the FAZ. (E) Selected pixel map of SCP excluding the FAZ. (F-H) Calculation of deep capillary plexus (DCP) vascular density (VD) excluding the FAZ using the Otsu auto-local threshold. (F) Original image excluding the FAZ. (G) Binarized image of DCP excluding the FAZ. (H) Selected pixel map of DCP excluding the FAZ. (I-K) Calculation of choriocapillaris porosity using the Phansalkar auto-local threshold. (I) Original image. (J) Binarized image of the choriocapillaris. (K) Flow void pixel map of the choriocapillaris.

For choroidal vascularity index (CVI) analysis, OCT B-scan images were processed using ImageJ based on the method described by Iovino C et al. [7] and Tellioglu A et al. [14] (Fig 1). First, the polygon tool was used to define the entire choroid as the region of interest, which was saved in the ROI manager. The image was then converted to 8-bit and binarized using the Niblack auto-local threshold (15 window radius) to differentiate the luminal area (LA) and stromal area (SA). The image was then converted to RGB, and LA was highlighted using the color threshold tool and added to the ROI manager. The total choroidal area (TCA) and LA were measured, and CVI (the ratio of LA to TCA) was calculated. SA was defined as the difference between TCA and LA.

For OCTA imaging, a 4.5 mm x 4.5 mm scan was used. The foveal avascular zone (FAZ) area and vessel density (VD) of the superficial capillary plexus (SCP) and deep capillary plexus (DCP) were analyzed, and flow voids in the choriocapillaris were detected to calculate choriocapillaris porosity (Fig 1). The FAZ area was manually outlined along the inner vascular boundary using ImageJ’s freehand selection tool, and the area was measured [15]. To analyze vessel density (VD) in the SCP and DCP, the Otsu auto-local threshold (15 window radius) was used for image binarization [16,17]. After excluding the FAZ area, VD (%) was calculated using the ‘Analyze Particles’ tool (≥1 pixel). Additionally, flow voids in the choriocapillaris images were detected to assess choriocapillaris porosity. The images were binarized using the Phansalkar auto-local threshold (15 window radius), and choriocapillaris porosity (%) was calculated using the ‘Analyze Particles’ function (≥1 pixel) in ImageJ [17,18].

### Statistical analyses

Statistical analysis was performed to compare the differences between the CME group and the non-CME group using the Mann-Whitney U test. The analysis was also conducted between the stages, dividing them into CME and non-CME groups, with comparisons made between early vs. moderate, moderate vs. advanced, and early vs. advanced stages using the Mann-Whitney U test three times to analyze intergroup differences. Additionally, Spearman correlation analysis was conducted within the CME group to examine which factors are related to visual acuity and cyst area. Statistical significance was defined as p < 0.05.

## Results

A total of 130 eyes from 130 patients with RP were included in the study, with 42 patients in the CME group and 88 in the non-CME group. Comparative analysis revealed several statistically significant differences between the two groups. The CME group demonstrated a significantly higher CMT compared to the non-CME group (p = 0.000). Conversely, the non-CME group had significantly higher SCT, TCA, SA, and LA (p < 0.05). Additionally, the foveal avascular zone in the superficial capillary plexus (FAZ_SCP) was significantly smaller in the CME group compared to the non-CME group (p = 0.022). When comparing disease stages, the CME group was more likely to be in the moderate stage of RP (47.62% vs. 26.14%, p = 0.015), while the non-CME group had more advanced cases (39.77% vs. 9.52%, p = 0.000) (Table 1).

**Table 1:** Comparison of RP patients with CME and without CME.

	With CME (n = 42)	Without CME (n = 88)	p-value
Age (yrs)	54.00 (42.50-62.50)	49.00 (33.00-61.75)	0.342
Sex (M/F)	19/23	43/45	0.700
Lens status			
Phakic/Pseudophakic	32/10	61/27	0.419
Underlying disease			
DM	2	4	0.956
HTN	9	12	0.261
Heredity			
AD	1	3	0.752
AR	1	6	0.296
VA (logMAR)	0.36 ± 0.51	0.57 ± 0.73	0.290
**CMT (µm)**	257.50 ± 104.98	171.40 ± 73.15	**0.000** ^*^
**SCT (µm)**	246.98 ± 87.31	294.52 ± 122.85	**0.043** ^*^
**TCA (mm**^**2**^)	3.82 ± 1.34	4.64 ± 1.98	**0.031** ^*^
**SA (mm**^**2**^)	1.53 ± 0.54	1.85 ± 0.76	**0.025** ^*^
**LA (mm**^**2**^)	2.30 ± 0.81	2.79 ± 1.22	**0.033** ^*^
CVI	0.60 ± 0.02	0.60 ± 0.03	0.780
EZ length (µm)	2828.66 ± 2226.29	2278.01 ± 2565.53	0.060
**FAZ_SCP (mm**^**2**^)	0.27 ± 0.12	0.42 ± 0.31	**0.022** ^*^
FAZ_DCP (mm^2^)	0.32 ± 0.17	0.47 ± 0.41	0.367
VD_SCP (%)	31.77 ± 2.49	31.79 ± 3.63	0.964
VD_DCP (%)	30.10 ± 3.45	30.41 ± 4.55	0.664
CC porosity (%)	39.44 ± 1.43	41.71 ± 7.55	0.824
Stage			
Early (n, %)	18(42.86%)	30(34.09%)	0.335
**Moderate (n, %)**	20(47.62%)	23(26.14%)	**0.015** ^*^
**Advanced (n, %)**	4(9.52%)	35(39.77%)	**0.000** ^*^

* p-value of <0.05 was considered as statistically significant.

RP = retinitis pigmentosa, CME = cystoid macular edema, DM = diabetes mellitus, HTN = hypertension, AD = autosomal dominant, AR = autosomal recessive, VA = visual acuity, logMAR = Logarithm of the Minimum Angle of Resolution, CMT = central macular thickness, SCT = subfoveal choroidal thickness, TCA = total choroidal area, SA = stromal area, LA = luminal area, CVI = choroidal vascularity index, EZ = ellipsoid zone, FAZ = foveal avascular zone, SCP = superficial capillary plexus, DCP = deep capillary plexus, VD = vessel density, CC = choriocapillaris

In both groups, visual acuity (VA) worsened in the advanced stages (p < 0.01). In the CME group, the cyst area was significantly larger in the advanced stage compared to the early stage (p = 0.041). CVI significantly decreased from the early to moderate stage (p = 0.015). Additionally, the foveal avascular zone in the deep capillary plexus (FAZ_DCP) increased from the moderate to the advanced stage (p = 0.025), while vessel density in the deep capillary plexus (VD_DCP) decreased from early to moderate stages (p = 0.036). Choriocapillaris (CC) porosity significantly increased in the advanced stage compared to the early stage (p = 0.030). In the non-CME group, patients in the advanced stage were significantly older compared to those in the early stage (p = 0.004), and a significantly higher proportion of patients had undergone cataract surgery in the advanced stage (p < 0.01). Both CMT and the CVI progressively decreased as the disease advanced (p < 0.05), and SCT significantly reduced in the advanced stages (p < 0.05). VD_SCP and VD_DCP showed a significant decline from early to moderate stages (p < 0.01), while FAZ_DCP and CC porosity increased in the advanced stages (p < 0.05) (Table 2).

**Table 2:** Comparison of RP patients by stage, divided into CME and non-CME Groups.

	Early (n = 48)	Moderate (n = 43)	Advanced (n = 39)	p-value (E vs. M/M vs. A/E vs. A)
**CME (Early n = 18, Moderate n = 20, Advanced n = 4)**				
Age (yrs)	50.39 ± 16.79	50.60 ± 17.57	58.50 ± 18.08	0.942/0.373/0.327
Sex (M/F)	8/10	10/10	1/3	0.735/0.370/0.485
Lens status				
Phakic/Pseudophakic	15/3	15/5	2/2	`0.535/0.326/0.160
Underlying disease				
DM	1	1	0	0.940/0.655/0.637
HTN	4	5	0	0.843/0.271/0.309
Heredity				
AD	0	1	0	0.343/0.655/1.000
AR	1	0	0	0.292/1.000/0.637
**VA (logMAR)**	0.22 ± 0.19	0.23 ± 0.22	1.62 ± 0.79	0.928/0.003^*^/0.003^*^
CMT (µm)	254.78 ± 40.80	246.50 ± 87.55	324.75 ± 292.84	0.174/0.699/0.217
SCT (µm)	251.67 ± 104.00	248.75 ± 80.01	217.00 ± 31.65	0.661/0.314/0.496
**Cyst Area (µm**^**2**^)	8391.94 ± 7297.96	16368.81 ± 25055.98	164897.10 ± 280957.02	0.704/0.104/0.041^*^
TCA (mm^2^)	3.94 ± 1.43	3.73 ± 1.39	3.79 ± 0.84	0.661/0.816/0.798
SA (mm^2^)	1.54 ± 0.59	1.50 ± 0.54	1.57 ± 0.36	1.000/0.699/0.670
LA (mm^2^)	2.39 ± 0.84	2.22 ± 0.85	2.21 ± 0.51	0.539/0.877/0.733
**CVI**	0.61 ± 0.01	0.59 ± 0.02	0.58 ± 0.03	0.015^*^/0.535/0.061
EZ length (µm)	4924.91 ± 1750.63	1507.77 ± 518.85	0	0.000^*^/0.002^*^/0.002^*^
FAZ_SCP (mm^2^)	0.27 ± 0.11	0.26 ± 0.14	0.38 ± 0.03	0.604/0.180/0.114
**FAZ_DCP (mm**^**2**^)	0.32 ± 0.18	0.28 ± 0.12	0.64 ± 0.09	0.716/0.025^*^/0.076
VD_SCP (%)	32.82 ± 2.59	31.04 ± 2.37	31.52 ± 1.26	0.102/0.655/0.236
**VD_DCP (%)**	31.83 ± 3.94	28.75 ± 2.71	30.63 ± 1.87	0.036^*^/0.297/0.693
**CC porosity (%)**	39.20 ± 0.81	39.29 ± 1.59	41.90 ± 0.52	0.775/0.074/0.030^*^
**Non-CME (Early n = 30, Moderate n = 23, Advanced n = 35)**				
**Age (yrs)**	42.47 ± 16.94	45.26 ± 19.03	54.83 ± 15.35	0.621/0.055/0.004^*^
Sex (M/F)	15/15	10/13	18/17	0.641/0.557/0.909
**Lens status**				
**Phakic/Pseudophakic**	24/6	20/3	17/18	0.508/0.003^*^/0.009^*^
Underlying disease				
DM	0	1	3	0.253/0.538/0.103
HTN	2	3	7	0.436/0.496/0.124
Heredity				
AD	1	1	1	0.849/0.763/0.912
AR	1	3	2	0.189/0.335/0.651
**VA (logMAR)**	0.15 ± 0.21	0.29 ± 0.49	1.12 ± 0.80	0.142/0.000^*^/0.000^*^
**CMT (µm)**	226.27 ± 38.60	195.04 ± 52.56	108.83 ± 59.72	0.007^*^/0.000^*^/0.000^*^
**SCT (µm)**	308.93 ± 105.61	339.57 ± 132.82	252.57 ± 119.67	0.647/0.007^*^/0.011^*^
TCA (mm^2^)	4.79 ± 1.61	5.11 ± 2.15	4.21 ± 2.11	0.774/0.103/0.111
SA (mm^2^)	1.86 ± 0.64	2.02 ± 0.81	1.73 ± 0.83	0.590/0.195/0.337
LA (mm^2^)	2.93 ± 0.98	3.09 ± 1.34	2.47 ± 1.29	0.844/0.066/0.056
**CVI**	0.61 ± 0.02	0.60 ± 0.02	0.58 ± 0.04	0.021^*^/0.031^*^/0.000^*^
**EZ length (µm)**	5422.12 ± 1633.86	1643.54 ± 594.12	0	0.000^*^/0.000^*^/0.000^*^
FAZ_SCP (mm^2^)	0.32 ± 0.12	0.34 ± 0.18	0.61 ± 0.47	0.849/0.118/0.060
**FAZ_DCP (mm**^**2**^)	0.26 ± 0.15	0.36 ± 0.24	0.84 ± 0.54	0.342/0.005^*^/0.000^*^
**VD_SCP (%)**	34.28 ± 3.27	30.13 ± 2.66	30.26 ± 3.23	0.002^*^/0.890/0.002^*^
**VD_DCP (%)**	33.79 ± 3.62	29.11 ± 2.59	27.38 ± 4.51	0.001^*^/0.198/0.000^*^
**CC porosity (%)**	39.06 ± 0.94	39.70 ± 3.68	47.12 ± 11.72	0.649/0.024^*^/0.012^*^

* p-value of <0.05 was considered as statistically significant.

RP = retinitis pigmentosa, CME = cystoid macular edema, E = early, M = moderate, A = advanced, DM = diabetes mellitus, HTN = hypertension, AD = autosomal dominant, AR = autosomal recessive, VA = visual acuity, logMAR = Logarithm of the Minimum Angle of Resolution, CMT = central macular thickness, SCT = subfoveal choroidal thickness, TCA = total choroidal area, SA = stromal area, LA = luminal area, CVI = choroidal vascularity index, EZ = ellipsoid zone, FAZ = foveal avascular zone, SCP = superficial capillary plexus, DCP = deep capillary plexus, VD = vessel density, CC = choriocapillaris

**Fig 2:**
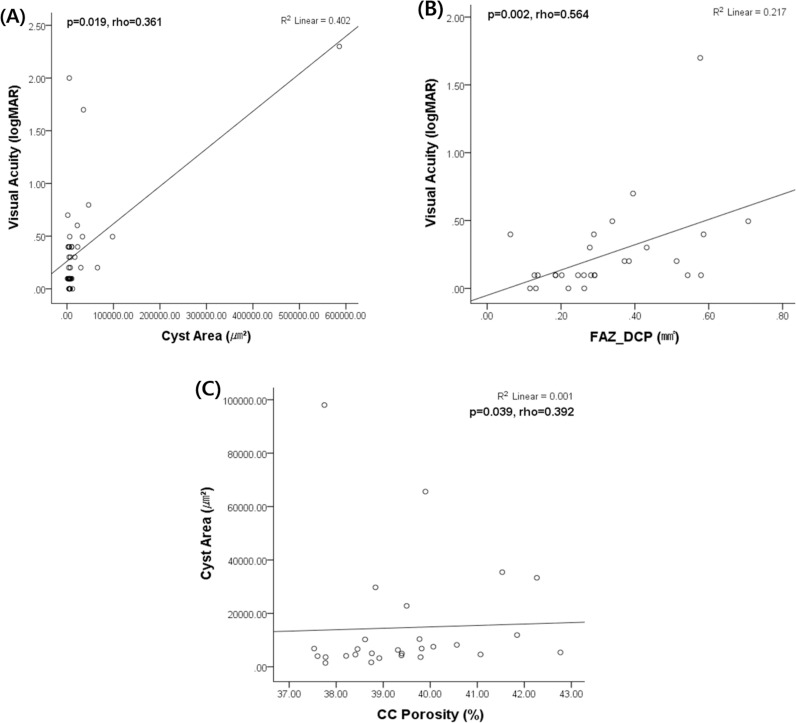
Optical coherence tomography (OCT) parameters correlated with visual acuity and cyst area in patients with RP-CME. (A) Positive correlation between Cyst Area and Visual Acuity (logMAR) (p = 0.019, rho = 0.361, R² = 0.402). (B) Positive correlation between FAZ_DCP and Visual Acuity (logMAR) (p = 0.002, rho = 0.564, R² = 0.217). (C) Positive correlation between choriocapillaris (CC) Porosity and Cyst Area (p = 0.039, rho = 0.392, R² = 0.001).

In the CME group, larger cyst area (p = 0.019, rho = 0.361) and wider FAZ_DCP (p = 0.002, rho = 0.564) were associated with worse visual acuity. Additionally, cyst area was positively correlated with CC porosity (p = 0.039, rho = 0.392) (Fig 2).

## Discussion

Compared to the non-CME group, the CME group exhibited higher CMT but lower choroidal factors, including SCT, TCA, SA, and LA (Table 1), suggesting a potential association with overall choroidal atrophy in CME patients. Additionally, the higher proportion of moderate-stage cases in the CME group and the greater prevalence of advanced-stage patients in the non-CME group (Table 1) suggest a potential relationship between CME and choroidal atrophy. To further elaborate on this, given that advanced RP is typically associated with significant retinal and choroidal atrophy, the lower choroidal parameters observed in the CME group, despite the non-CME group having more advanced-stage patients, imply that CME itself may be associated with choroidal atrophy, independent of disease stage. However, while the reduction in choroidal parameters (SCT, TCA, SA, and LA) in the CME group suggests alterations in the choroid, it does not necessarily confirm choroidal atrophy. We consider this reduction to be a necessary but not sufficient condition for choroidal atrophy, as various factors, including hemodynamic alterations and inflammatory responses, may also contribute to these changes. Therefore, further investigations, including histopathological analysis and functional assessments, may be needed to accurately characterize choroidal atrophy in CME patients. In contrast to our findings, another study analyzing choroidal characteristics in RP-CME [7] reported increased SCT in RP-CME patients. They hypothesized that RP-CME might result not only from Müller cell dysfunction but also from thickened choroid due to localized increased blood flow caused by intraocular inflammation. However, that study included patients with epiretinal membrane (ERM), with an ERM prevalence of 43.3% in the CME group and 29.4% in the non-CME group (p = 0.07). The mean ERM grade was similar between the CME group (1.6 ± 0.8) and the non-CME group (1.4 ± 0.6), with no statistically significant difference (p = 0.90). Although the prevalence of ERM between CME and non-CME groups was not statistically significant (p = 0.07), this borderline value, coupled with other research suggesting ERM can increase SCT [19], makes it difficult to completely rule out the potential influence of ERM on SCT increase in CME patients.

The significantly smaller FAZ_SCP observed in the CME group may be attributed to the lower proportion of advanced stage patients compared to the non-CME group (Table 1). On the other hand, the study by Mansour AM et al. on CME associated with gyrate atrophy reported that the superficial FAZ area was larger in CME patients compared to the normal control group [20]. In our study, the smaller FAZ_SCP observed in the CME group may be due to macular cysts compressing the FAZ, leading to a reduction in its size.

Regarding CVI, both Iovino C et al. [7] and Tellioglu A et al. [14] reported lower CVI in cases with CME compared to those without. Our study found no significant difference in CVI between CME and non-CME groups, which may be due to the higher proportion of advanced stage patients in the non-CME group potentially lowering the group’s CVI and masking the CVI reduction effect of CME presence.

We excluded pathologies such as ERM and vitreomacular traction that could affect retinal/choroidal parameters. Our results suggest that CME may be associated with choroidal atrophy regardless of disease stage, indicating that RP-CME could be more related to the degree of RPE dysfunction and choroidal damage caused by chronic low-grade intraocular inflammation due to oxidative stress, rather than the extent of disease involvement. This inflammation likely stems from outer retinal degeneration initiated by rod cell deterioration.

Current understanding suggests that RP-CME is caused by a complex interplay of blood-retina barrier breakdown, Müller cell dysfunction, RPE dysfunction, and inflammation, all secondary to outer retinal degeneration [6–8]. Further research is needed to elucidate the precise sequence of these events.

In the stage-wise analysis of the CME group, visual acuity tended to deteriorate further in the advanced stage (Table 2). Additionally, cyst area appeared larger in the advanced stage compared to the early stage. Although not presented in a separate table or figure, analysis of CME prevalence by stage revealed no difference between early and moderate stages (p = 0.387), higher prevalence in moderate compared to advanced stages (p = 0.000), and higher prevalence in early compared to advanced stages (p = 0.004). We speculate that in the advanced stage, overall retinal atrophy reduces the space available for edema formation, potentially explaining the lower observed CME prevalence in this stage. Similarly, another study [8] reported a tendency for cyst area to decrease as the outer retinal layers degenerate in advanced stages of retinal degeneration.

Interestingly, while our study found lower CME prevalence in the advanced stage, cyst area was significantly larger. This suggests that although CME occurrence is less frequent in highly degenerated retinas, when it does occur, it may be more extensive. We hypothesize that in advanced RP, reduced retinal structural stability, impaired RPE pump function, and choroidal atrophy may contribute to larger cyst formation when it does occur, despite the overall reduction in space due to retinal atrophy. However, it should be noted that our advanced stage CME group consisted of only four patients, which may have influenced these results. Further research is needed to confirm these findings.

During disease progression from the early to moderate stage, a decline in CVI and VD_DCP was observed, while in the transition from moderate to advanced stages, the FAZ_DCP showed an expansion. Additionally, compared to the early stage, increased choriocapillaris porosity in the advanced stage suggests progressive choroidal alterations as the disease worsens. Meanwhile, the small number of advanced-stage CME patients (n = 4), with only two suitable for OCTA analysis, may have contributed to the lack of statistical significance in certain analyses.

Interestingly, VD and FAZ in the SCP remained relatively stable across disease stages. This stability, despite disease progression, suggests a potential compensatory mechanism to the SCP. Given that CME primarily occurs in the inner nuclear layer (INL) and outer plexiform layer (OPL) [21], and the DCP is located near these layers [22], CME is more likely to compress the DCP. Previous studies have suggested that intraretinal cystoid spaces may exert mechanical effects on retinal structures. Murakami T et al. proposed that foveal cystoid spaces could compress parafoveal capillaries, leading to transient nonperfusion [23]. Karahan E et al. further reported that intraretinal cysts might induce axonal compression, contributing to axoplasmic stagnation [24]. Additionally, Berlin A et al. provided histological evidence of intraretinal fluid compressing the outer nuclear layer in neovascular AMD [25]. These findings suggest that mechanical compression by intraretinal fluid may influence retinal microvascular structures, potentially contributing to alterations in vascular density and function. Given these considerations, we hypothesize that compensatory mechanisms in the SCP, which is connected to the DCP in the retinal vascular network [26], may maintain its stability across stages. A similar compensatory vascular mechanism between DCP and SCP has been proposed in Fabry Disease [27].

Previous studies [10,28] reported that in RP progression, the DCP, being closer to photoreceptors, is affected earlier than the SCP, and vessel density reduction precedes FAZ enlargement. Our findings align with this, showing early DCP vessel density reduction and FAZ enlargement occurring in the moderate-advanced progression.

In contrast to the CME group, the non-CME group exhibited typical retinal and choroidal changes associated with disease progression, as widely reported in previous studies (Table 2) [10,29–31]. Visual acuity deteriorated further in the advanced stage, alongside a progressive reduction in CMT. SCT also decreased in the advanced stage, while CVI showed a gradual decline as the disease progressed. Examining VD in the SCP and DCP, we observed a noticeable decline during the early-to-moderate disease progression. On the other hand, the FAZ in the DCP exhibited enlargement substantially in the advanced stage. Similar to the aforementioned study by Oh R et al. [10], VD reduction occurred early, while FAZ enlargement manifested later. The FAZ in the SCP, considered to be affected last in the disease sequence, showed no significant differences between stages in the non-CME group. Meanwhile, the p-value for FAZ_SCP enlargement between early and advanced stages was 0.060. Although this was not statistically significant, a larger sample size might have revealed a meaningful difference between the groups. Moreover, similar to the CME group, choriocapillaris porosity showed a notable increase in the advanced stage.

Intriguing differences emerged in the stage-wise comparison between CME and non-CME groups. Firstly, the CME group showed no significant differences in SCT across stages, whereas the non-CME group exhibited thinning in the advanced stage. Secondly, VD_SCP remained stable across stages in the CME group but decreased in the early-moderate progression in the non-CME group. In summary, the CME group demonstrated consistently thin SCT regardless of stage, with VD_DCP decreasing in the early-moderate progression while VD_SCP remained stable across stages.

Correlation analysis within the RP-CME group revealed that larger cyst area and a wider FAZ_DCP were associated with worse visual acuity (Fig 2). Another study by Ruff A et al. [8] investigated the relationship between visual acuity and cyst area, revealing results that both resemble and differ from our findings. They reported an initial two-line decrease in visual acuity associated with cyst formation, which is similar to our observation that cyst areas have a negative impact on visual acuity. However, they observed no further decline in visual acuity as cyst size continued to increase over time. Additionally, although the explanatory power was minimal (0.1%), larger choriocapillaris porosity was associated with a larger cyst area (Fig 2). While the contribution of choriocapillaris porosity to cyst area enlargement appears minimal, we speculate that choroidal microvasculature atrophy during RP progression may impede fluid outflow, potentially leading to cyst area expansion. Taken together, CME in RP patients may act as both a detrimental factor and a compensatory trigger. As a detrimental factor, larger cyst areas were associated with worse visual acuity in the CME group. However, the observed maintenance of VD_SCP across disease stages suggests that CME may also function as a compensatory trigger, potentially inducing vascular responses that help sustain VD_SCP. Regarding whether the maintenance of VD_SCP in CME is beneficial, we believe that it is certainly more favorable than its reduction. While VD_SCP maintenance is not an absolute determinant of visual function, it may contribute to the stability of retinal perfusion, which could be advantageous for preserving the neurovascular unit.

This retrospective study has several limitations. It was conducted in a racially homogeneous Korean population, which may limit the generalizability of the findings to other ethnic groups. Additionally, factors such as environmental influences, healthcare accessibility, and socioeconomic status were not considered. Selection bias may have occurred due to the exclusion of patients with poor image quality or those who did not undergo OCT examination. We limited our analysis to patients imaged with the Topcon DRI OCT Triton^®^ at their initial visit, which may have affected the observed proportion of CME in RP patients. Patients imaged with the Heidelberg Spectralis HRA+OCT^®^ at their initial visit were excluded to facilitate angiography analysis. In addition, we acknowledge that circadian choroidal thickness variation was not controlled as a confounding factor due to the retrospective nature of our study. To address this, we additionally analyzed the OCT examination time (08:36–17:22) and found no significant difference between the CME (12:49 ± 2:44, range: 08:52–16:42) and non-CME (12:32 ± 2:53, range: 08:36–17:22) groups (p = 0.720). Furthermore, no significant differences were observed across disease stages within the CME group (early: 12:35 ± 2:39, moderate: 13:06 ± 2:51, advanced: 12:31 ± 3:10; p > 0.05 for all comparisons). However, in the non-CME group, a significant difference in examination time was found between the moderate (11:38 ± 3:03) and advanced (13:14 ± 2:39) stages (p = 0.017). We considered whether this difference could influence our findings through circadian choroidal thickness variation. However, based on our results, we believe that the impact of stage progression on the observed reduction in SCT and CVI (Table 2) is far greater than that of examination time. As supporting statement, SCT and CVI also significantly decreased in the non-CME group when comparing early vs. advanced stages, even though no significant examination time difference was observed between these groups (p = 0.221; early: 12:24 ± 2:53, advanced: 13:14 ± 2:39). Additionally, the circadian variation observed in healthy individuals may potentially decrease in RP patients due to structural and vascular alterations. Based on these assumptions, the trend of reduced SCT and CVI in the advanced stage is likely to persist as the disease progresses. Nonetheless, the potential influence of circadian variation cannot be entirely excluded; therefore, further studies are needed. Additionally, the selection of the eye with the greater CMT in cases of bilateral CME may not sufficiently consider the natural history and asymmetry of RP, which could influence the study findings. Although we excluded patients who had undergone ocular surgery within the past 6 months, we cannot entirely rule out the possibility of late-onset Irvine-Gass syndrome. Given the elapsed period after cataract surgery in the 10 pseudophakic patients within the CME group (mean: 9.17 years, range: 8 months–22 years), this possibility appeared unlikely. However, as some cases have been reported even years after surgery, it remains a potential consideration. Furthermore, multiple comparison adjustments were not applied when performing Mann-Whitney U tests across the three groups, which may have increased the likelihood of type I errors. We acknowledge this limitation and note that careful interpretation is required. Further studies are needed for validation. The limited number of patients who underwent next-generation sequencing (NGS) genetic testing precluded a comprehensive analysis of genetic factors. In addition, RP staging of this study was based on structural parameters rather than functional criteria such as visual acuity or visual field loss. While structural assessment via OCT provides an objective measure of disease progression, incorporating functional evaluations in future studies may offer a more comprehensive understanding. Moreover, since our study focused only on initial presentation data, it does not account for intermediate or final follow-up results. As a result, changes in CME status over time could not be assessed. Some patients with CME may not have initially had CME, while others without CME may have previously experienced it. The average follow-up period for these patients before inclusion was 2.6 years; however, this study does not reflect longitudinal changes. Future studies with extended follow-up are needed to better understand CME progression in RP. The small number of advanced stage CME patients may have limited statistical significance for some variables, potentially obscuring expected outcomes. Additionally, in unilateral CME cases, further analysis comparing choroidal thickness in the affected and unaffected eyes within the same patient would be valuable. This could provide additional insights into whether choroidal changes are localized to the CME-affected eye or represent a systemic feature of RP progression.

In conclusion, our findings suggest that RP-CME may be associated with choroidal atrophy and RPE dysfunction, which could contribute to fluid accumulation in the retina, independent of disease stage. These structural changes may lead to vascular alterations, where SCP and DCP exhibit potential regulatory mechanisms in response to retinal edema and ischemia. The maintenance of vessel density in SCP across disease stages could reflect a compensatory response to DCP compression caused by intraretinal fluid accumulation. Additionally, in RP-CME, larger cyst area and FAZ_DCP were correlated with worse visual acuity, potentially serving as indicators of visual function. Further research is needed to clarify the interplay between RPE dysfunction, choroidal atrophy, and microvascular changes in RP-CME, as understanding these mechanisms may help guide future therapeutic strategies.
